# Can tonic motor activation be the magical elixir for restless legs syndrome?

**DOI:** 10.1093/sleep/zsad230

**Published:** 2023-09-06

**Authors:** Joseph Andrew Berkowski, Brian B Koo

**Affiliations:** ReLACS Health, Ann Arbor, MI, USA and; Department of Neurology, Yale University, New Haven, CT, USA

Restless legs syndrome (RLS) in its severe, chronic form often requires lifelong medical therapy. However, in recent times, the delivery of long-term treatments for RLS has been fraught with obstacles and drawbacks. Chief among these impediments has been the study, marketing, broad usage, and then over-prescription of dopamine agonists, as these medicines cause a worsening of RLS over months to years in the phenomenon of augmentation [1].

Augmentation is the obvious example, yet treatment challenges of severe RLS extend well beyond the dopamine agonist conundrum. Gabapentin enacarbil is the only other FDA-approved medication for the treatment of RLS, yet its prescription is limited due to its expense and a lack of third-party payer coverage. It belongs to the alpha-2-delta ligand class of drugs along with gabapentin and pregabalin that form a consensus first-line treatment group of medications[1]. Though this class is effective in treating RLS for many patients, side effects such as drowsiness and dizziness often limit their use, and their potency is likely further reduced in patients living with RLS, who have previously experienced augmentation. Intravenous iron is another important first-line RLS therapy [2]. Time and again, RLS providers hear from their patients who have not been able to find a provider who will arrange for an iron infusion. This is likely due to an erroneous belief that iron given intravenously is dangerous and potentially due to perceived complexity of ordering intravenous iron. As if these issues were not enough to impede access to adequate treatment for the individual with chronic RLS, the single most effective medical therapy for severe, refractory RLS, opioid medications, became widely overprescribed for a number of other conditions, leading to a national opioid crisis. As a result, many providers are unwilling to prescribe opioids for severe RLS even though opioids have been shown to be effective and stable at low doses in patients with RLS [3].

With both physicians and patients feeling burned by dopamine agonists and having reservations about the current recommended therapies, non-pharmacological treatments represent the holy grail, the possibility of delivering considerable symptomatic relief without the systemic effects of ingesting a pharmaceutical compound. A variety of noninvasive devices including those delivering vibration or compression to legs have been on the market over the years but none, to this point, has demonstrated efficacy in a randomized, sham-controlled trial.

In this month’s issue of *SLEEP*, two studies provide optimism for the future of non-pharmacological treatment for RLS. Bogan et al. report positive findings from a sham-controlled device trial on tonic motor activation (TOMAC), while Roy et al. present the continued efficacy of TOMAC in an open-label extension study [4, 5]. The wearable device, which delivers TOMAC, is applied under the knees of both lower extremities, delivering nerve stimulation to the peroneal nerve (common fibular n.). After initial calibration, the device attempts to deliver a comfortable impulse leading to tonic motor activation of the innervated anterior tibialis muscle, thus providing afferent feedback to the central nervous system to shut off RLS symptoms. The premise is to mimic active movement like ambulation, which canonically relieves RLS symptoms. The device may be worn prior to bed and even during sleep and, unlike walking, can provide symptom relief while the patient stays in bed, allowing for sleep maintenance. In these studies, TOMAC could be used for 30 minutes in up to four sessions per 24-hour period.

The study by Bogan et al. compares the TOMAC device to sham in 133 patients with severe RLS for 4 weeks in stage 1. In stage 2, participants were unblinded, and all received the active treatment for an additional 4 weeks. The primary endpoint of Clinical Global Impressions-Improvement (CGI-I) showed 45% were responders with the active device compared to 16% with sham (difference = 28%; 95% CI: 14% to 43%; *p* = 0.00011). A secondary endpoint of the IRLS Score showed a −3.4 reduction in IRLS compared to sham (95% CI: −1.4 to -5.4; *p* = 0.00093). Only 2 of the 68 participants in the active group withdrew due to adverse effects (discomfort and administration site irritation) in stage 1 and no participants withdrew from the open-label extension. In stage 2, in those from the original active treatment group, CGI-I increased from 45% to 61% and IRLS Score improved from −7.2 to −8.7. Additionally, the Medical Outcomes Study Sleep Scale (MOS-I & MOS-II) showed statistically significant improvement in the active versus sham group with continued improvement in stage 2.

Roy et al. report the open-label extension of the original trial with 103 patients for an additional 24-week period of treatment in which participants were divided into treatment and non-treatment (control) groups after the initial 8-week trial. The CGI-I responder rate was 72.7% in the treatment group versus 13.6% in the non-treatment group (*p* < 0.0001) after 24 weeks. Similarly, IRLS Score was improved by −11.3 (95% CI: −8.8 to −13.9) compared to −5.4 (95% CI: −3.7 to −7.2; *p* = 0.0001 for treatment v. control). No patients withdrew due to adverse events and all device-related adverse events were classified as grade 1 (mild).

These studies demonstrate that TOMAC of the peroneal nerve can be efficacious compared to sham with short-term use in a refractory RLS cohort, the first noninvasive device to do so in a rigorous trial. The 4-week open-label extension and then the second study with additional 24-week extension give credence to the long-term efficacy of this device. The authors do report positive trajectory of study metrics over the course of the 24-week extension, even compared to the first 8-week time point, suggesting a trend toward continuous improvement of the condition over time-related to the device. Certainly, some of this can be from the sham/placebo effect of unblinding, as well as, another typical study phenomenon like the Hawthorne effect and natural improvement from severe extremes, but with the low number of only mild adverse effects, having multiple direct and indirect factors leading to improved RLS and sleep quality cannot hurt.

Though it is true that sham control is not as fool-proof as placebo control within medication trials, the thoroughness of this approach and the results in several RLS and sleep domains compared to sham should provide optimism for this technology. However, TOMAC will now have to withstand the tests of real-world use when available to the market. The combined results suggest efficacy up to 32 weeks with minimal risk of adverse effects is a great start. The field was completely fooled by trials of dopamine agonists, most of which were completed in much less than one year, as augmentation occurs cumulatively across individuals over many years. Noninvasive nerve stimulation on the surface, however, seems to have less potential for central nervous system adaptation or the emergence of significant adverse effects as has been seen with dopamine agonists. TOMAC is certainly not intended to eliminate the need for important pharmacological treatments for RLS including alpha-2-delta ligands, IV iron, and opioids. It does, however, add a lower-risk option to the armamentarium, and one that potentially can be used simultaneously with pharmacotherapy. Amidst sleepy times in enthusiasm for both clinical management and research in RLS, TOMAC takes a step toward stimulating renewed interest in the development of new treatments for RLS.
